# Report of Discussions at the Ohio State Dental Society, Dec., 1 and 2, 1868

**Published:** 1869-01

**Authors:** 


### Proceedings of Societies.

### REPORT OF DISCUSSIONS AT THE OHIO STATE DENTAL SOCIETY, DECEMBER 1 AND 2, 1868.

### THE MEANS OF CONTROLLING THE FLOW OF SALIVA DURING OPERATIONS.

DR. BUTLER: I suppose it is well understood by all that the great difficulty we have to contend with in filling teeth is the saliva, or fluids of the mouth, and the great difficulty in controlling them so that we may be able to make successful operations, especially upon the inferior teeth. Of course, men have their peculiar modes of doing this, and stick to them with a great deal of tenacity. These are the rubber dam, the napkin, the duct compressor, &c.

Some will condemn the use of one mode for all cases. I have tried nearly all the different appliances that have been brought before the profession, and notwithstanding some claim to the contrary, there are some cases that I am unable, with any thing that I have seen to control the saliva, so that I can make such fillings as I would desire.

With these general statements, I would simply say that I do not think that any one mode is the best in all cases. I have not had much practical experience in the rubber dam, but would say that in many cases it seems to be the only thing that will protect the case so that you may operate upon the teeth. The greatest objection I have to it is that it requires considerable ingenuity and skill to use it in such a manner as to answer the purpose for which it is designed. Unless the preparation is made of a good article, and made nicely, it is liable to tear. There is, also, a difficulty in turning the edge down, which is the great point. After it has passed over the tooth to be operated upon, it must be turned down and the silk thread tied above it so that it will not slip.

In my own practice, I resort to the use of small napkins, and believe that the less complicated the appliances for this purpose the better. There is a sort of repugnance to putting any of these clap-traps in the mouth. They seem to excite the flow of saliva, and you actually defeat the attempt to control the moisture.

The use of large napkins, I think, is inadmissible. They are in the way of the operator, and fill the mouth so much, and annoy the patient so that they do more harm than good, and then you can not change them so readily as small ones. There are a great many operations we are called upon to make now-a-days, which it is impossible to complete without changing the napkin several times. The manner of introducing and consolidating the gold requires time, and the cavity must be free from moisture.

In the first place—to give a little detail of the procedure—In proximal cavities, I would use the wedge of wood, putting it in each side of the tooth I am going to work upon, that shuts off any flow of moisture at the base of the cavity, and gives a view of the lower margin, and protects the gum in the operation of packing the gold and finishing the filling. If there was only this advantage in the wedge, that is sufficient to balance all the inconvenience of its introduction. In those cases where the teeth are a little feeble and loose, and where there is that spongy condition of the gums, where it is difficult in suppressing the flow of moisture, I find by wedging up several teeth along, you succeed quite well in shutting off the moisture which is so great an annoyance.

Then, in using napkins, I would use no other than those that can be readily folded in a convenient shape to place under the tongue and outside the labial surface. Using smaller ones outside than inside, so the tongue will not be forced back in swallowing. There are some patients who are not able, or do not know how to swallow if there is any thing in the mouth, and with them it will be difficult to control the moisture. By placing the napkin in on the side where you are going to work, and allow it to come around perhaps two-thirds of the way, it may be easily changed when it becomes saturated. But this is a matter that very few seem to have become master of—just handling those little compresses so as to accomplish the object.

I would simply state in addition to the use of these small napkins—going back to the rubber dam in cases I can not control with the napkins; I put in the rubber dam, and in some cases I believe it is necessary. This flow of moisture must be controlled though it demands time, if we would have the operation such as we would expect to get good results from. There are many, I know, that come short of obtaining the best results, from the fact of being unable to control the moisture. You may talk about submarine filling, but I would not resort to it if it could be avoided. That is the last resort, and then only an imperfect operation can be made. The large gold fillings that are demanded at our hands, require different manipulations throughout from those of ten or fifteen years ago. There are a great many fillings now made that ten years ago we would have thought impossible, especially in the inferior teeth. There is not much difficulty in controlling the moisture on the superior teeth and making operations, but it is with the inferior teeth that the great difficulty occurs.

Dr. Spellman: I am constantly using the rubber dam, but it is often very annoying. Sometimes I am on the eve of giving it up, because it slips off when I get it placed. When the rubber dam is properly adjusted, you can draw the mouth up as you wish, and go into the reception room and chat with patients, though the filling is not completed. When properly put on, it is impossible for the water to pass between that and the tooth. In filling the inferior teeth I regard it as an indispensable article. When operating on a bicuspid, I very often, in order to succeed with the rubber dam, place it over three teeth and operate on the middle one. It is almost utterly impossible to put the rubber over the inferior cuspid in such a way as that the water will not leak through. In such cases, I always have cut up in readiness, pieces of spunk, and if it leaks, and they become wet, I change them as Dr. Butler changes his napkins. You will see at once, when the spunk is saturated and should be changed. With regard to the inferior incisors, it is very difficult to apply the rubber dam, yet it can be done by taking a piece of silver wire, or if you have not that, very small wrapping wire, and bringing forward, hold it with a blunt instrument and crowd it below the crown of the tooth, then pass your rubber down as near the wire as possible, then slipping the silk over this twice after, and with a fine instrument, slip the edge of it over that wire. It is so liable to slip up, but with this wire it can be held down. So far as the upper teeth are concerned, I use it a great deal, though I don't regard it as indispensable any where except for the inferior teeth.

In filling the superior bicuspids, the wedge is my dependance. But we find that the gums seem to secrete a kind of watery mucus that is not naturally a product of the salivary glands, and when engaged in an operation of filling, you will discover it is becoming moist. This spunk, if pressed into the interstices between the teeth in such cases, will always show you when it is time to change it. When there is danger of water, I never fill approximal cavities, or attempt to do it, without pressing into the interstices between the teeth pieces of spunk in this way, and if the wedge passes far enough above the base of the cavity so as to put a very thin piece of spunk in, I prefer it, for, although you dry it off the wood well, the water will work through it, and the first pellets will become a little dampened. I would rather have every point clean and dry, for when you come to filling the upper margin, you can not polish or finish off with so nice and smooth a surface as if it had not got wet or dampened by contact with the wood. I use bibulous paper where Dr. Butler uses napkins.

I don't know whether it is better or as good. [A member. "It is good, though expensive."] I use it because I thought it was not so expensive. A quire of this paper will last a good while. It is rather a delicate tissue paper, and not so rough to the mouth as supposed. It will contain a great deal of water. Formerly, in using it, I took and folded it closely, one layer over another, folding it compactly and rendering it hard, supposing that the more I got in the more water it would contain. The paper is capable of great expansion, and will contain water in proportion as you allow it to expand. Afterwards, I found that half the amount of paper would hold as much water, and folded more loosely, it was not so harsh or offensive to the soft velvet-like tissue it is laid upon.

Dr. BUFFETT: I can not tell you how to control the flow of saliva. I can tell how I attempt to do it. I confess I have not received very much benefit from the rubber dam. It has been a failure in my hands to a great extent. I use the holder sometimes for holding the cheek back, and in cases which I consider difficult, as the inferior teeth, sometimes have the strap to pass around the neck and head and held by the patient. That I consider an indispensable appliance, and on the inferior teeth I depend almost entirely on napkins, and occasionly on the tongue holder. Instead of the napkin I use linen cloth, called diaper, containing considerable starch, so as to contain a certain amount of stiffness; if it is washed it is sometimes too flimsy. I cut it into pieces from an inch to four inches square. Placing these small pieces and pressing them under the tongue, and the large napkin under the end folded in a strip. You can change either the larger or smaller napkins as many times as you wish.

The fewer things we put in the mouth the better we can operate, and with more ease to ourselves and patients. It depends a great deal on the firmness, as you may say, of the operator. If you determine to control the flow of saliva, and let the patient understand what you intend, you will be more likely to succeed. But if you go at it indifferently and undecided, your patient will think there is going to be a great deal of trouble, and they will get excited, so that you can not make a good operation. Even if I think there is danger I don't tell them. If I fail I try again.

Dr. Herriott said he tried to control the condition of the patient. He applied something to the teeth after they were excavated, applied white wax and sealed up the cavity, and delayed the filling a day or two. Had this engagement arranged beforehand, then, when the patient returned, there was no excited condition of the patient, as immediately after the excavation of the teeth, when the flow of saliva would be greater than if the patient had been quiet for a day or two.

Dr. N. W. Williams, for the last few weeks, had been using the new duct compressor—Smith's—the part that passes under the chin has a latteral motion, also that which passes inside. His way of using it is, to cut out a piece of spunk something in the shape of a half moon and place on the ducts, and then a small napkin laid round inside of the teeth under the tongue, placing the duct compressor on that, and pressing down as tightly as it would admit of without irritating the muscles. He had succeeded better in controlling the flow of saliva with that apparatus than any thing he has tried before.

Before he got that, he had succeeded pretty well by the use of the napkin and spunk. He had, by directing the patient to crook the finger and place the napkin in the mouth and hold it, succeeded in cases where he had failed before.

He thought spunk controlled the flow of saliva better even than the use of napkins, for, as Dr. Spellman says, we can, by using it, generally tell when there is approaching danger. He had often succeeded by having a piece of spunk near at hand and applying it. But by applying the spunk under the tongue over the saliva ducts he had found particular advantage.

### MECHANICAL DENTISTRY.

Dr. Berry said: I hoped Dr. McClelland would have been here to elaborate somewhat his invention of "Rose Pearl," as a base for teeth. I have been for years anxiously hoping we should get something that would deliver us from the intolerable nuisance of rubber, but I am not quite certain that we have it yet. I hope rose pearl may prove successful.

The use of rubber has been a great detriment to our profession. As a base for teeth it does not afford sufficient strength; and especially is this the case in partial sets. Also, from the manner of securing the teeth on them, breakage of the teeth is much more than on metallic bases.

Rubber has let into the profession a set of graceless, unprincipled, miserable quacks, who, with an overwhelming stock of impudence, proclaim themselves dentists, after studying long enough to enable them to mount teeth on rubber, as they may, spending fewer weeks at it probably than would be required of years to properly prepare them for practice. It is among those that the "Ten-dollar-a-set" advertisers are found.

But for rubber we should not have been annoyed by these charlatans, nor the people cursed with them. It is strange that every member of the community does not understand when a Dental practitioner offers his services for almost nothing, he is the best judge of their value, and that of artificial teeth, the best they can get are none too good.

Rubber comes in as a cheap, mean, inferior substitute for something better—this is all we can say for it. We should endeavor to elevate the standing of mechanical Dentistry, and urge on our patients the importance of having their artificial teeth constructed on strong, durable material, and paying for them an honest Christian compensation.

Dr. SPELLMAN said while the rubber advent has been a very unfortunate one, in one aspect of the case, it has been a very fortunate advent in another aspect. I believe that the Dental profession to-day stands higher than it would have stood had it not been for the rubber advent. We will go back to the time it made its advent, in 1854, perhaps—at least that was the time we commenced to make rubber work in the State of Ohio. Every one present will bear me witness that every laboratory in Ohio was well supplied with all the appliances necessary to do artificial work. If you called upon a Dentist, you were more likely to find him in his laboratory than in his office. There was a great amount of labor performed in the laboratory. That class of men who had ability were patronized in this specialty; that is in continuous gum work, gold, silver, &c., and it required so much skill that none but men of talent could succeed. But the rubber advent changed that and drove them from the laboratory to the operative room. The profession has turned its attention to preserving the natural organs. So far as the rubber advent has conduced to this end, it has been a blessing to the profession and a blessing to the world. But the rubber itself is a miserable failure. It has brought into the profession a class of men that have taken to the operating room—men with no qualification, as I have said. What impudence for men that have but one prominent feature of mental qualification—that is consummate impudence and dishonesty. The honorable members of the profession, scorning to put themselves on a level with such men, have done as little as possible in putting in artificial teeth, so as to meet the wants of their patients and have adhered to that business for which they went into the Dental profession. I say, then, that the profession has been elevated by this rubber advent, and it has drawn a line of demarkation between these empirics and worthy men in the profession. It has enabled us to take a step in advance, and make a stride in the direction of conservative Dentistry, that in 1854 was never contemplated. Now, mark, I attribute this to the rubber advent; but, while I am making these remarks, I would not have any one construe them for a moment so as to think I am an advocate of the rubber. I detest it. I detest it because very many people will class us with these persons that are doing now most of the rubber work in our section of the country. It is there done mostly by quacks, and I presume it is so in other sections of the country. I believe we all understand that if we have any new ideas to throw out which may be of advantage to the members, that we feel it to be our duty to do so. Let me ask the Association to follow me back again for a few moments to that period prior to this rubber advent. We sought at one time to obtain a metal for dies, or swedges, as they were sometimes called, that would not shrink in cooling. We had preparations of tin, type metal, &c., and sought to overcome the shrinkage in the metal with the view of getting a plate that was exactly the size of the mucous membrane of that portion of the mouth which should be covered by it. A few years' experience, it will be remembered, showed that the theory was impracticable, and not only impracticable, but incorrect; that really those traces made on the casts or swedges did shrink. That is my experience and the experience of many. I know it is the experience of some of our best men. Profiting by that experience since I went into the rubber work, I have brought into my practice a method that secures that end. I do it in this way: The experience of every one present will bear me witness that a ring upon a finger at some times, in some conditions of the flesh or tissue, is such that it will slip off easily, and at other times it can hardly be removed. Now, what is the difference? Cold shrinks or diminishes animal tissue, and heat expands it. When you put into the mouth the plaster of Paris, the very moment that same result commences to take place, heat is evolved that acts upon the membrane of the mouth and expands it. You get an impression of the mouth that is not an exact counterpart of what the mouth was before you put the plaster into it. It is very true that it is with great difficulty that you can remove that impression, and a great many argue that the impression must be a good one, because it adheres so tightly to the mouth, and is removed with great difficulty. This is not the reason why it adheres with such tenacity. It is because the soft tissues were expanded by the heat of the plaster, making a perfect adaptation to the surfaces of the one with the other, and you get the pressure of the atmosphere of fifteen pounds to the square inch. Negatively, that enlarging the tissues here crowds the plaster, or compresses it, and the result is that you have not a perfect impression of the mouth. My plan to overcome this difficulty is this: I take a tumbler of ice water and give it to my patient, and tell the patient to hold it in the mouth until it gets benumbed and chilled, and while this is being done I go into the laboratory and prepare my impression cup, which had been previously prepared somewhat with reference to that mouth. I prepare the plaster in no hurry, letting the patient take the icy water, and when it is warm throwing it into the spittoon and filling the mouth again. I then put in my plaster and I get, as every one present will admit, a smaller impression than if taken with the mouth in its normal condition. I succeed much better in that way, and have made sets of teeth that the patients could not remove from the mouth, and after they had worn them three or four days, came to my office, claiming that I had cheated them by putting teeth in that were not intended to be removed. Another method I resort to sometimes to overcome that expansion in the mouth is this—let me, however, go back a little before I give it. A professor in Philadelphia has published a treatise upon the manipulation of rubber. He tells you that you should not varnish the plaster impression, but that you should coat it over with a thin coat of soap or soapy water, where the soap has dissolved with some little consistence; then put in your plaster, and claims that you get a cast more firm and a better impression. I admit that it is so, but deny that it is necessary. I take the cast or impression and give it, if time, two or three coats of gum shellac, taking care to apply it evenly, with a view of shrinking the cast I get from that impression, and make it smaller.

Dr. BERRY said the expansion of the plaster in setting renders our casts larger, as we all knew. He asked Dr. Spellman if, in varnishing, he varnished as much in the roof as the labial surface, with a view to make the cast less.

Dr. SPELLMAN replied that it depended entirely on the condition of the mouth; if the alveoli ridges were hard and unyielding he varnished equally; if they were soft and yielding he not only varnished, but laid on two or three thicknesses of tin foil, and put the varnish on two or three thicknesses. He went upon the general theory, in his operations, that we make our plates to fit only when they are pressed up and require but little pressure to put them in their place.

Dr. KELSEY said sometimes we think we get a good fit with a plaster cast, because it holds with such tenacity to the mouth, but sometimes when we make a plate in that way it is not what we expect, and is not satisfactory. He wished to speak of the lower plates more particularly. He had had some difficulty sometimes with the lower plates rising up from the mouth, and had adopted a plan lately by which he found he got the suction more satisfactorily than in any other way. It was suggested by having a temporary plate made for a gentleman some sixty years of age. It would move about, and when he would bite upon one side it would tilt up. As it was a temporary case he wished to experiment on it, he took a file and filed a small groove around the whole arch, and, putting it in his mouth, found that it had remedied the defect.

Dr. Berry: How far from the ridge did you file this groove?

Dr. Kelsey: Right on the top where the alveoli process ought to be, though there really was none. In a case where he had a good deal of difficulty he remedied it by taking a little square drill and drilling holes through the plate, when it held firmly. In taking it off he found each of these holes filled with the mucous membrane, which held it tightly.

Dr. Herriott took impressions of the mouth with as little plaster of Paris as possible, and dropping them in water let them remain until the plaster absorbed all the water it would contain. After it became crystalized he took the impression, and around the mouth and at the arch of the palate pared off a little. There was as much difference in mouths as in faces, but in that way he was able to get very satisfactory fits. He had as a case, recently, that of a set of teeth of James E. Murdock. In his profession the great changes made in his mouth and face, bringing into exercise all the muscles of the mouth and face, had developed it beyond that of any mouth he had ever attempted to set teeth in. His teeth had first been put in by Dr. J. D. White, of Philadelphia, and next by a Dentist of Cincinnati. While at Cincinnati he threw them out in his lecture, but was enabled to get them back with but few of the audience knowing any thing about it.

He said he was very much annoyed and had to be very careful with them. Dr. H. said he had left his office, not thinking they would do any better than they had. He went to Cleveland, and Dr. Horton went to see him, and the doctor reports the case a success. Dr. H. said, in his case, here moved quite an amount around, after he had taken from the impression so that he didn't think it would hold in the mouth, but after it was in the mouth awhile he found they became very firm.

Dr. KELSEY mentioned a case he had of congenital cleft palate, of a lady about forty-five years of age. The teeth had all been extracted, except the posterior bicuspid teeth above. The cleft was very large, reaching far forward. He took an impression in wax in the usual way, by filling the cup very full and letting the wax extend up into the fissure as far as it would, taking particular care to press it upward in the back portion of the cleft, and reaching back with a pair of plyers, drawing it pretty nearly to its normal condition, while the wax in the mouth was very soft. In pressing it in that way of course a portion of the wax extended up into the fissure, which he brought down as well as he could to the point he wished the plate to be. He poured the plaster in the usual way, though before pouring in the plaster he trimmed down the wax where it was needed. Then he took particular pains, after having it vulcanized, to make it antagonize on the teeth below. Thus vulcanized, it was pressed up into the fissure, so that after it was worn a day or two it produced a little irritation, it was then necessary to shorten that portion and round it down a little, to make it more agreeable to the patient. That was made in one solid piece, without any attempt at making a palate, but, to give her own words, she was "so pleased with the arrangement that she could not sleep that night." Before, she could not blow out a candle; it seemed that the cavity was so large that the air passed through the nostrils, and some out of the mouth, in such a way that she could not blow a steady stream. But now she is enabled to blow out a candle, suck her food down without any effort, and has improved her speech very much. Dr. Kelsey also related his successful treatment of the case of a fractured jaw of a boy, who was injured by a fire engine. The appliances he used, and the manner of adjusting them, can not be well given here so as to be fully understood.

Dr. Keely mentioned a case of an old gentleman seventy-five years of age, who was kicked by a horse and thrown against some logs, by which his jaw was broken between the front incisors. These were the only teeth he had that seemed to have any firmness. He had an incisor and two bicuspids on one side, none of which were firm, and no teeth on the other side nor none above. Dr. K. spoke of the difficulty in taking an impression in this case, and exhibited the manner in which it was done, illustrating the process by using the President as if he were the patient, in describing the appliances used. The jaw of the old gentleman got well, though his face was left somewhat disfigured.

Dr. G. W. Dunn desired to make some statements with regard to a specialty of his, as some of them were aware he had made a specialty of porcelain work. He thought he might safely say that it had made such an advance within a year or a year and a half past as it had never made before. He thought if the profession would look to their interest as they ought, they would find that they need not make so many miserable plates as they are making upon rubber. He was satisfied that in the hands of skillful operators, porcelain work can now be manufactured much nicer and better than any other work that has ever been presented to the profession. When he said this he did not say it egotistically; he said it because he believed it, and could produce the evidence to prove his assertion. The material, as now manufactured, can be furnished to operators in a plastic condition, capable of being moulded by the operator at his will, and the teeth placed in such position as artistic skill would dictate, giving, at the same time, the whole scope, shape and range desired for the teeth and color for the gums to the hands of the operator, which he thought could not be said of any other style of work. It appeared to him, if the profession would take the opportunity to examine cases that had been worn for twelve or fourteen years past, and then cases that are now being made by men who have not had long experience, but who can take hold of the work as now furnished them, that there would not be much rubber work made, but that they would lay it aside. He could give the testimony of hundreds, where they had worn rubber and where they had porcelain properly fitted in their mouths, where it would be almost impossible to get them to wear rubber, or any metallic substance, in the mouth. It appeared to him the profession are overlooking their interests in neglecting to investigate and understand the merits of porcelain work.

Dr. W. E. Dunn had for a long time felt convinced that if the profession could understand the merits of this style of work, they would be glad to give it their attention. With his experience he felt that he should not do his duty unless he expressed his confidence in it. He did not think it was because the profession have any design or wish to overlook a matter of this important character, but probably because it has been brought to their attention in its imperfect manipulation in the hands of some who had not the advantages they ought to have had. He found that persons who had given it their attention have been convinced that it has not the objections they had supposed it had. The satisfaction of patients who were using it is remarkable. He would be gratified if members of the Association and others, would give attention to the cases that might come under their notice, and examine the success of this work.

